# Validation of the REM sleep behavior disorder screening questionnaire in the Czech population

**DOI:** 10.1186/s12883-019-1340-4

**Published:** 2019-06-04

**Authors:** Jitka Bušková, Pavla Peřinová, Eva Miletínová, Petr Dušek, Evžen Růžička, Karel Šonka, David Kemlink

**Affiliations:** 1 Department of Neurology and Center of Clinical Neurosciences, 1stFaculty of Medicine, Charles University, Kateřinská 30, 12000 Praha 2, Czech Republic; 2 Department of Sleep Medicine, National Institute of Mental Health, Klecany, Czech Republic

**Keywords:** Rapid eye movement sleep behavior disorder, Screening for synucleinopathy, Self administered questionnaire

## Abstract

**Background:**

Idiopathic rapid eye movement sleep behavior disorder (iRBD) affects 1–2% of people over 60 years of age and presents a high risk of developing a neurodegenerative disorder from the group of synucleinopathies, such as Parkinson’s disease, dementia with Lewy bodies and multiple system atrophy. Therefore, screening tools are needed. In 2007, the rapid eye movement sleep behavior disorder screening questionnaire (RBD-SQ) was developed and has been translated into several languages. The aim of study was to assess the validity and reliability of the Czech version of the RBD-SQ in a mixed population of sleep clinic patients, supplemented by healthy volunteers and RBD patients.

**Methods:**

Participants included 81 iRBD patients, 205 patients with other sleep disorders (obstructive sleep apnea, insomnia, restless legs syndrome and periodic limb movement disorder, other parasomnias, or central hypersomnias including narcolepsy) and 20 healthy volunteers.

**Results:**

The mean RBD-SQ score in the iRBD patients was 9.4 ± 2.8 points, and in the non-RBD group it was 4.5 ± 3.0 (*P* < 0.0001). Receiver -operator analysis yielded an area under the curve of 0.864, suggesting good diagnostic performance of the scale. When using a cut-off value for positivity of 5 points, sensitivity was 0.89 and specificity was 0.62.

**Conclusions:**

The Czech version of the RBD-SQ is a sensitive tool for screening for iRBD patients and helps to identify subjects for complete clinical workup.

## Background

Rapid eye movement (REM) sleep behavior disorder (RBD) is defined as a repetitive occurrence of vocalization and/or complex motor behavior and impaired muscle atonia during REM sleep [1]. In the general population, the prevalence of RBD is 0.38–2.1% [2, 3]. The idiopathic form of RBD (iRBD) is an important risk factor for later development of neurodegenerative disorders from the group of alpha-synucleinopathies [4, 5]. Patients suffering from iRBD are suitable candidates for neuroprotective therapy; therefore, effective screening of these patients is important. However, a definite diagnosis of RBD is made by means of night polysomnography, which is costly, time consuming and, thus, can only be performed on a limited number of individuals. Therefore, a highly sensitive and sufficiently specific detection of potential RBD patients is a logical step in the detection of RBD cases on a population scale. The first line among methods for such screening are self-administered questionnaires. The first specific method for RBD screening - RBD-SQ (REM sleep Behavior Disorder Screening Questionnaire) was published in 2007, and consists of 13 questions answered either “yes” or “no”, with each positive answer scoring 1 point [6]. RBD-SQ has also been validated in several languages outside of Europe, including Turkey, Japan, Korea and China [7–11]. Another screening tool, the REM sleep Behavior Disorder Questionnaire-Hong Kong (RBDQ-HK) [12], was published three years later, and in addition to actual symptoms of RBD it includes items for evaluating the occurrence of symptoms for one year prior to administration. Other questionnaires for RBD screening include the Mayo Sleep Questionnaire [13, 14], the Innsbruck REM sleep behavior disorder inventory [15] and a single question screen for rapid eye movement sleep behavior disorder [16].

RBD-SQ is the most broadly used tool and the aim of our study was to validate the Czech version of the questionnaire.

## Methods

A total of 306 participants of two sleep centers in Prague were included in the study and were then divided into three groups: 1) 205 adult patients of the mixed population (excluding iRBD), 2) 20 healthy volunteers and 3) 81 patients with RBD.

The mixed sleep population patients in the first group were further sub-divided into five sub-groups: 1) Obstructive sleep apnea (OSA), 2) restless legs syndrome and periodic limb movements in sleep (RLS/PLMS), 3) insomnia, 4) parasomnias - both non-REM and REM (other than iRBD), and 5) central hypersomnia (including narcolepsy).

All of the participants, including healthy volunteers, were examined by videopolysomnography. REM sleep without atonia (RWA) was scored according to the ICSD3 criteria [1] with the suggested montage including the upper extremities [17], and was detected in all of the iRBD patients, and also in four patients with parasomnias other than RBD, in three healthy volunteers, and in one RLS, one narcolepsy and one OSA patient.

Cognitive impairment was excluded in all of the participants including healthy controls using the validated Czech version of Montreal Cognitive Assessment (MoCA) [18], and all underwent a clinical neurological examination. None of participants from the iRBD group met the diagnostic criteria for Parkinson’s disease or Lewy body dementia and none had a history of any other neurological or psychiatric disorder. In addition, the iRBD patients previously participated in our web based survey, which is described elsewhere [19]. At the time of completing the questionnaire, all of the participants were free of any psychoactive medication and had signed the informed consent. The study was conducted according to the declaration of Helsinki and was approved by the Ethics Committee of the General University Hospital in Prague.

Double reverse translation of the RBD-SQ from Czech to English and German was performed and the resulting Czech version is present in Table 1. All of the participants stated Czech language as their mother tongue and all completed this version. The participants were instructed to answer the questions according to their lifelong occurrence of the mentioned symptoms and also include an opinion of their bed partners, if applicable.

**Table 1:** The Czech wording of the RBD-SQ [6] - Sample copy - do not use without permission

	Otázka	Odpověď
1.	Někdy mívám velmi živé sny.	Ano/Ne
2.	Mé sny často mívají agresivní náboj nebo obsahují živý děj.	Ano/Ne
3.	Obsah snu většinou odráží mé chování ve spánku.	Ano/Ne
4.	Jsem si vědom/a toho, že ve spánku pohybuji končetinami.	Ano/Ne
5.	Stalo se už, že jsem (téměř) zranil/a mého spolunocležníka nebo sám/sama sebe.	Ano/Ne
6.	Stává se/stalo se, že jsem ve spánku (ze sna):	
6.1.	Mluvil/a, křičel/a, nadával/a, hlasitě se smál/a	Ano/Ne
6.2.	Pohyboval/a prudce (až bojovně) končetinami	Ano/Ne
6.3.	Prováděl/a bezúčelná gesta a složité pohyby jako např.: mávání, salutování, odhánění komárů, pád z lůžka, apod.	Ano/Ne
6.4.	Shodil/a předměty nacházejí se v okolí lůžka, např.: Stolní lampu, knihu, brýle, apod.	Ano/Ne
7.	Stává se, že mě tyto pohyby probudí.	Ano/Ne
8.	Po probuzení si většinou dobře pamatuji obsah snu.	Ano/Ne
9.	Můj spánek bývá často narušený.	Ano/Ne
10.	Léčil/a jsem se/léčím se pro onemocnění nervového systému (např. cévní mozková příhoda, úraz hlavy, Parkinsonova nemoc, syndrom neklidných nohou, narkolepsie, deprese, epilepsie, zánětlivé onemocnění mozku), pro jaké?	Ano/Ne

RBD-SQ contact information and permission to use: Mapi Research Trust, Lyon, France. Internet: https://eprovide.mapi-trust.org

Abbreviations: *RBD* Rapid eye movement sleep behavior disorder, *RBD-SQ* Rapid eye movement sleep behavior disorder screening questionnaire

Statistical analyses were performed using the Dell Statistica (a data analysis software system), version 13.0. (software.dell.com).Non-parametric tests were used (Mann-Whitney U-test - MWU, Kruskal-Wallis ANOVA) for intergroup comparisons of the RBD-SQ scores.

## Results

The mean RBD-SQ score was 9.4 ± 2.8 in RBD patients, and 4.5 ± 3.0 in the whole non-RBD sub-group including the healthy volunteers (*P* < 0.0001 MWU test). The mean RBD-SQ score in individual sub-groups was as follows: OSA 4.0 ± 3.2, RLS/PLMS 3.8 ± 3.0, insomnia 4.3 ± 2.5, non-RBD parasomnias 6.8 ± 2.9, hypersomnias 5.0 ± 3.0 and healthy volunteers 3.5 ± 2.7.All of the sub-groups significantly differed from the iRBD group. The RBD-SQ had a Cronbach’s alpha value of 0.843, showing good consistency of the test [20]. All demographic data are summarized in Table 2.

**Table 2:** Demographic data in individual sub-groups

		Age (y)	Males
Sub-group	n	Mean ± SD	(% of n)
iRBD	81	61.2 ± 8.3	89
Healthy volunteers	20	45.2 ± 15.2	40
Insomnia	63	44.5 ± 17.3	44
OSA	52	57.2 ± 15.4	69
RLS/PLMS	38	53.6 ± 17.5	61
Non-RBD parasomnia	22	46.2 ± 16.2	55
Narcolepsy/hypersomnia	29	35.2 ± 12.4	48

Abbreviations: *OSA* Obstructive sleep apnea, *RBD* Rapid eye movement sleep behavior disorder, *RLS/PLMS* Restless legs syndrome and periodic limb movements in sleep

The lowest sensitivity was found for item 10 (42.5%), which ask for the history of neurological diseases. Further items with low sensitivity values were items 9 (48.8%), 4 (51%) and 8 (58.8%). In these items, the participants were asked to recall disturbed sleep, limb movements, and the content of their dreams, respectively.

The highest sensitivity was found for items 1 (93.8%), 6.2 (93.8%) and 6.1. (91.3%), which asked about vivid dreams, sleep vocalization and violent and/or sudden limb movements in sleep, respectively. However, item 1 (vivid dreams) had a low specificity, with as many as 80% of the healthy controls answering positively. Patients in the RLS/PLMS sub-group frequently answered positively to item 7, 8 and 9 (awakening due to limb movements, dream recall and disturbed sleep, respectively). In item 4 (awareness of limb movements), patients with RBD gave significantly more positive answers than the RLS/PLMS patients (*p* = 0.003). Diagnostic profile of individual questions is presented in Table 3.

**Table 3:** Sensitivity, specificity and % of positive answers of individual items

Item	Sensitivity	Specificity	AUC	iRBD	Healthy volunteers	Insomnia	OSA	RLS/PLMS	Non-RBD parasomnia	Narcolepsy/Hypersomnia
	%	%		%	%	%	%	%	%	%
				*n* = 79	*n* = 20	*n* = 63	*n* = 52	*n* = 38	n = 20	*n* = 29
1	93.8	23.3	0.585	93.7	80	79.4	65.4	63.2	100	89.7
2	86.3	55.2	0.707	86.4	30	52.4	26.9	34.2	70	65.5
3	80	79.8	0.799	79.8	10	20.6	15.4	15.8	50	17.2
4	51	79.8	0.655	60.8	5	9.5	34.6	31.6	35	37.9
5	80	86.5	0.833	79.8	10	6.3	7.7	7.9	65	13.8
6.1	91.3	52.5	0.719	91.1	55	36.5	40.4	34.2	85	69
6.2	93.8	78	0.859	93.7	5	9.5	26.9	21.1	60	27.6
6.3	85	83.9	0.844	84.8	10	9.5	13.5	15.8	60	10.3
6.4	57.5	82.5	0.700	58.2	15	15.8	13.5	7.9	55	17.2
7	74.8	72.2	0.730	73.4	15	25.4	28.9	21.1	60	27.6
8	58.8	42.2	0.505	58.2	65	68.3	51.9	55.3	50	51.7
9	48.8	47.1	0.479	49.3	30	69.8	48.1	55.3	60	34.5
10	42.5	68.6	0.556	41.8	15	38.1	30.8	21.1	35	37.9

Abbreviations: *AUC* Area under curve, *OSA* Obstructive sleep apnea, *RBD* Rapid eye movement sleep behavior disorder, *RLS/PLMS* Restless legs syndrome and periodic limb movements in sleep

The receiver-operator curve of the total score yielded AUC 0.864, suggesting good overall diagnostic performance of the RBD-SQ. When using a cutoff value of 5 points as proposed by the questionnaire’s authors, the sensitivity was 95.1% (95% confidence interval 88.0 to 98.1%) and specificity 59.6% (95% confidence interval 53.0 to 65.8%).

There were more male participants in the study than female (63.4% of the whole study sample). When analyzing positive responses to individual questionnaire item between males and female in the whole sample, there were significant differences for the following items: item 3 (positive in 24.6% women versus 42.5% men, *p* = 0.00175), item 5 (positive 16.4% versus 39.4%, *p* = 0.00003), item 6.1 (positive 50% versus 64.3%, *p* = 0.015), item 6.2 (positive 21.8% versus 50.3%, *p* = 0.00001), item 6.3 (positive 18.2% versus 43.5%, *p* = 0.00001), and item 7 (positive 30.0% versus 45.6%, *p* = 0.008). In the iRBD sub-group, we found no differences in distribution of responses between men and women.

The range of answers to item 8 (dream recollection) was not significantly different among any of the tested sub-groups, not even between the RBD patients and the healthy controls. In addition, for item 9 (disturbed night sleep), the RBD patients provided similar answers to the OSA, insomnia, RLS and parasomnia patients.

## Discussion

Our study proved that the Czech version of the RBD-SQ has sufficient validity and reliability for screening for RBD, and represents the first such study among Slavic languages.

The total score of 5 points represents the best cut off value for discrimination between iRBD patients and the other participants, including the healthy controls. We replicated the findings of the original publication: sensitivity 97.3% (the value lies within the 95% CI of our findings), and specificity 45.9% (slightly lower than our result) [6]. The same cut of value of 5 was replicated also in the Chinese and Japanese studies [9, 10]. The Italian validation study suggested raising the cutoff to 8, whereby increasing the specificity to 78%, but lowering the sensitivity to 84.2% [8].

The highest total score was in the iRBD group, and the lowest was in the group of healthy volunteers, with all of the other sub-groups having intermediate values, but still significantly different from the RBD group. This finding corresponds to unspecific positive responses common for other sleep problems, such as vivid dreaming, awareness of limb movements or disturbed sleep.

When analyzing the diagnostic accuracy of the individual items of the RBD-SQ, we confirmed lower specificity of item 1, 8, 9 and 10 similar to the previous publications [6, 8]. Positivity of item 1 was found in many other conditions than RBD, suggesting that vivid dreaming is also common across sleep pathologies. Recollection of these dreams in item 8 and disturbed night sleep in item 9 share the same characteristics. The Italian validation study suggested keeping item 10 as a clinically relevant indicator of possible false positive screening or a secondary form of RBD, but not to include it in the overall score [8]. Our data confirmed that this question had a moderate specificity (68.6%) in our set of iRBD patients.

RBD is characterized by behavior reflecting dream content and such motor activity may be dangerous for the patient and/or the bed-partner [21]. These symptoms are covered by items 5,6.2,6.3 and 6.4 and show the highest specificity and sensitivity, which is also in accordance with the original publication of the RBD-SQ [6].

Men are more frequently affected by RBD, usually it presents itself after the age of 60y [22]. In our sample, we found intersex differences in the way the items of the RBD-SQ were answered, and significantly in the case of items 3, 5, 6.1–6.3 and 7, suggesting that enacting dream content in women is generally less aggressive and less dangerous [23]. However, when analyzing only the iRBD sub-group, intersex differences are no longer significant, partly because of the low number of women (*n* = 9) with iRBD in our sample and the subsequent low statistical power.

Our study had several limitations. Despite being asked to include information from bed-partners, most patients completed the questionnaire in the sleep clinic, when their partners were not present. The healthy volunteers were relatively younger and their number was smaller. Moreover, all of the iRBD patients were older compared to other sub-groups and all had an idiopathic form of RBD.

However, the use of the RBD-SQ represents the first and broadest screening step in diagnostic workup for RBD. Therefore, it is important to know the diagnostic accuracy of this tool and expect false positive and false negative results based on the given application. For screening purposes, false negative results should be avoided as much as possible as they may lead to patients not being diagnosed. On the other hand, false positive screening may lead to costly and time-consuming polysomnography, which may turn out to be negative. This phenomenon occurs in at least 16% of healthy volunteers [24]. In addition, the use of multiple different RBD screening questionnaires may lead to conflicting results [25].

## Conclusions

The Czech version of the RBD-SQ represents a validated and reliable screening tool for the detection of RBD, with diagnostic accuracy similar to the original publication of the German version. It can be administered to a population of patients visiting a general practitioner or specialist (sleep clinic, neurology) and may help in indicating the requirement for further investigation and polysomnography.

## Data Availability

The datasets used and analyzed during the current study are available from the corresponding author upon reasonable request.

## Abbreviations

ANOVA: Analysis of variance
AUC: Area under the curve
iRBD: Idiopathic form of the RBD
MDS-UPDRS: Movement disorder society Unified Parkinson’s Disease Rating Scale
MoCA: Montreal cognitive assessment
MWU: Mann-Whitney U-test
OSA: Obstructive sleep apnea
RBD: Rapid eye movement sleep behavior disorder
RBD-SQ: RBD screening questionnaire
REM: Rapid eye movement
RLS/PLMS: Restless legs syndrome and periodic limb movements in sleep
RWA: REM sleep without atonia
